# Schwann cell durotaxis can be guided by physiologically relevant stiffness gradients

**DOI:** 10.1186/s40824-018-0124-z

**Published:** 2018-05-09

**Authors:** Elisabeth B. Evans, Samantha W. Brady, Anubhav Tripathi, Diane Hoffman-Kim

**Affiliations:** 1 Department of Molecular Pharmacology, Physiology, Brown University, Providence, Rhode Island, 02912 USA; 2 Center for Biomedical Engineering, Brown University, Providence, Rhode Island, 02912 USA; 3 Carney Institute for Brain Science, Brown University, Providence, Rhode Island, 02912 USA; 4 Center to Advance Predictive Biology, Brown University, Providence, Rhode Island, 02912 USA

**Keywords:** Schwann cell, Durotaxis, Peripheral nerve regeneration, Gradient, Band of Büngner, Morphodynamics

## Abstract

**Background:**

Successful nerve regeneration depends upon directed migration of morphologically specialized repair state Schwann cells across a nerve defect. Although several groups have studied directed migration of Schwann cells in response to chemical or topographic cues, the current understanding of how the mechanical environment influences migration remains largely understudied and incomplete. Therefore, the focus of this study was to evaluate Schwann cell migration and morphodynamics in the presence of stiffness gradients, which revealed that Schwann cells can follow extracellular gradients of increasing stiffness, in a form of directed migration termed durotaxis.

**Methods:**

Polyacrylamide substrates were fabricated to mimic the range of stiffness found in peripheral nerve tissue. We assessed Schwann cell response to substrates that were either mechanically uniform or embedded with a shallow or steep stiffness gradient, respectively corresponding to the mechanical niche present during either the fluid phase or subsequent matrix phase of the peripheral nerve regeneration process. We examined cell migration (velocity and directionality) and morphology (elongation, spread area, nuclear aspect ratio, and cell process dynamics). We also characterized the surface morphology of Schwann cells by scanning electron microscopy.

**Results:**

On laminin-coated polyacrylamide substrates embedded with either a shallow (∼0.04 kPa/mm) or steep (∼0.95 kPa/mm) stiffness gradient, Schwann cells displayed durotaxis, increasing both their speed and directionality along the gradient materials, fabricated with elastic moduli in the range found in peripheral nerve tissue. Uniquely and unlike cell behavior reported in other cell types, the durotactic response of Schwann cells was not dependent upon the slope of the gradient. When we examined whether durotaxis behavior was accompanied by a pro-regenerative Schwann cell phenotype, we observed altered cell morphology, including increases in spread area and the number, elongation, and branching of the cellular processes, on the steep but not the shallow gradient materials. This phenotype emerged within hours of the cells adhering to the materials and was sustained throughout the 24 hour duration of the experiment. Control experiments also showed that unlike most adherent cells, Schwann cells did not alter their morphology in response to uniform substrates of different stiffnesses.

**Conclusion:**

This study is notable in its report of durotaxis of cells in response to a stiffness gradient slope, which is greater than an order of magnitude less than reported elsewhere in the literature, suggesting Schwann cells are highly sensitive detectors of mechanical heterogeneity. Altogether, this work identifies durotaxis as a new migratory modality in Schwann cells, and further shows that the presence of a steep stiffness gradient can support a pro-regenerative cell morphology.

**Electronic supplementary material:**

The online version of this article (10.1186/s40824-018-0124-z) contains supplementary material, which is available to authorized users.

## Background

Peripheral nerve injuries often result in significant disability and greatly reduced quality of life from persistent neuropathic pain. Further, clinical consequences of peripheral nerve injuries are long-lasting, given that the highest incidence occurs most commonly in younger healthy patients [1]. Better clinical nerve repair treatments are needed, as functional recovery in patients with current treatments is often unsatisfactory and off-site complications arise at secondary surgical sites. Physical and functional impairments following injury are the result of incomplete or aberrant nerve regeneration across the nerve defect, in which the proximal nerve end either entirely or partially fails to reconnect to its original innervation target. In contrast, successful reinnervation is dependent upon directed migration of morphologically-specialized Schwann cells across the injury site. Once Schwann cells migrate into the defect and assemble into an aligned elongated structure, severed axons can regenerate, using this transient structure for directional guidance. If Schwann cells fail to either migrate across the nerve defect or establish a direct trajectory for axonal regrowth, the process of functional nerve regeneration will not occur.

Although several groups have developed biomaterials that present various guidance cues including chemotactic, haptotactic, and topographical gradients to enhance nerve repair through directed migration of Schwann cells [2], the current understanding of how the mechanical environment influences Schwann cell migration remains largely understudied and incomplete. Recent reports, including studies from our group, have begun to characterize the mechanosensitivity of Schwann cells [3–5]. Specifically, we previously observed that migratory Schwann cells variably alter their traction forces on uniform substrates tuned to different discrete stiffnesses found within the peripheral nervous system. Given that the post-injury nerve environment is mechanically dynamic and heterogeneous, in this study we cultured Schwann cells on substrates embedded with stiffness gradients to determine whether Schwann cells can display durotaxis, directed cell migration in response to a gradient of increasing stiffness [6].

The presence of mechanical gradients has previously been described in neurodevelopmental tissue niches, physiologic niches where the processes of both directional migration and morphological specialization are ubiquitous [7]. Focal adhesion kinase, which is necessary for durotaxis [8], also mediates specific physiological processes in Schwann cells, including the induction of a pro-migratory phenotype following nerve injury [9] and differentiation [10]. Both morphologic specialization of repair Schwann cells [11] and their directed migration across the nerve defect [12] are two concurrent processes that are independently critical in order for peripheral nerve regeneration to be successful. Altogether these studies informed the following hypotheses: stiffness gradients can (1) influence Schwann cell morphology and (2) provide a directional cue for their migration.

Our results indicate that Schwann cells can alter their cell morphology and migratory phenotype in response to a stiffness gradient. Notably, Schwann cells were able to directionally migrate, guided by stiffness gradients as shallow as 0.04 kPa/mm, which is a unit change across length of more than one order of magnitude less than shallow gradients previously evaluated in other durotaxis studies [13]. Unlike durotaxis reported in other cells adherent to substrates of different and varying stiffnesses, Schwann cells did not increase their speed or directedness as a function of the stiffness gradient steepness, however the gradient slope was an important parameter to elicit an elongated cell morphology. This study provides the first evidence for Schwann cell durotaxis and further defines stiffness gradient design parameters optimal to support directed migration and cell elongation, Schwann cell-specific behaviors displayed during the sequential phases of the peripheral nerve regeneration process.

## Methods

### Polyacrylamide (PAA) substrate fabrication, functionalization, and surface characterization

Polyacrylamide substrates were prepared through a photoinitiated polymerization process, according to an established protocol described elsewhere [13, 14]. Briefly, degassed solutions of acrylamide (AAm) and bis-acrylamide (Bis) were polymerized with 0.5% Irgacure-2959 (Ciba 1-[4-(2-hydroxyethoxy)-phenyl]-2-hydroxy-2-methyl-1-propane-1-One), under a UV light box (254 nm) for 3.5 min. Substrates were fabricated between an activated 25 mm hydrophilic glass coverslip for attachment and a hydrophobic treated glass slide. A grayscale printed mylar transparency was placed between the UV light source and glass-PAA set-up for mechanical gradient patterning. For mechanically uniform substrates, a uniformly patterned 0 or 70% grayscale mask was used. For gradient substrates, a radial gradient grayscale mask (ranging from 0% at periphery to 70% at center) was used, to generate a substrate with a more compliant center and increasing stiffness towards the outer perimeter. Pre-polymer weight per volume concentrations were 4% AAm (0.15% Bis) and 10% AAm (0.225% Bis) respectively for the shallow and stiff gradients. Hydrogel surfaces were activated with the crosslinker N-sulfosuccinimidyl-6-(4’-azido-2’-nitrophenylamino) hexanoate (Sulfo-SANPAH, Thermo Scientific Pierce) and covalently modified with 0.2 mg/mL laminin (Thermo Fisher) for cell attachment.

### Mechanical characterization of substrates

Rheological data was acquired with a controlled-stress rheometer (AR-2000N, TA Instruments) using an 8 mm diameter stainless steel parallel plate geometry. To characterize the experimental gradient substrates, PAA substrates were prepared from the two different pre-polymer solutions with varying acrylamide-Bis concentration ratios and polymerized with UV light transmitted through different grayscale photomasks (from 0-70% in steps of 10%). Substrates corresponded to the stiffer substrate periphery (0%), more compliant substrate center (70%), and gradient substrate positions in between (10-60%). Experiments were performed in triplicate on independently fabricated substrates in hydrated conditions. Oscillatory rheometric tests were carried out at 25° C and with a compressive normal force between 0.16-0.27 N maintained. This corresponds to compressive stresses between 3.1 kPa and 5.4 kPa, which is within physiological range. For each substrate condition, strain sweeps (0.1-10%) at a constant frequency of 1 Hz (6.28 rad/s) were first performed to confirm that subsequent collected data fell within the linear viscoelastic region. Frequency sweep tests at 1% strain were performed over a range of 1 to 100 rad/s. The shear storage modulus (G ^′^), which describes elastic resistance, is reported for data acquired at 1 Hz. For comparison with other studies reporting Young’s elastic modulus (E), the corresponding E was calculated using the equation, E = 2G ^′^(1+ *ν*), where G ^′^ is the shear storage modulus as measured by the rheometer and *ν* is Poisson’s ratio, assumed to be 0.457 for polyacrylamide [15].

### Schwann cell culture

Schwann cells isolated from adult rat sciatic nerve (generous gift from Dr. Mary Bunge, University of Miami, Coral Gables, FL) were maintained in Dulbecco’s modified Eagle’s medium containing 10% fetal bovine serum, 4mM L-glutamine, 100 *μ*g/mL penicillin and 100 *μ*g/mL streptomycin, supplemented with 2 *μ*M forskolin (Sigma Aldrich), 10 *μ*g/mL bovine pituitary extract (Sigma Aldrich), and 2 *μ*M heregulin (Sigma Aldrich). Cells used were between passage 3 and 5. Dissociated Schwann cells were plated onto laminin-coated PAA substrates at a sparse density of 150 cells/cm^2^ to minimize cell-cell interactions.

### Timelapse microscopy

One-hour after cells were seeded onto PAA substrates, sequential phase-contrast images were collected with a Nikon Eclipse TE2000-S microscope, connected to a Hamamatsu Orca-ER camera and Orbit shutter control, equipped with a software-controlled motorized stage and enclosed with a custom humidified chamber to maintain 37° C and 5% CO_2_. Fields of view were randomly chosen for each substrate and images were collected every 12 min over a 24 h period.

### Cell-material surface characterization

Following timelapse observation, PAA substrates were prepared for physical surface examination under scanning electron microscopy (SEM). Samples were incubated in Karnovsky’s fixative overnight, rinsed twice with 0.1M sodium cacodylate buffer, incubated in 1% osmium tetroxide in 0.1M sodium cacodylate buffer for one hour, rinsed thrice with dH_2_O, incubated in 1% thiocarbohydrazide in dH_2_O for one hour, rinsed thrice with water, incubated with 0.5% osmium tetroxide in dH_2_O for one hour, dehydrated in ascending graded ethanol solutions for twenty min each (20-100% ethanol in 10% steps), critical point dried with liquid CO_2_, and sputter coated with 100 Å gold-palladium. Samples were examined with a Hitachi S-2700 SEM operated at an acceleration voltage of 8kV, with samples tilted at an angle of incidence of 20 degrees.

### Cell staining and epifluorescent imaging

Cells on substrates were fixed with 4% paraformaldehyde in phosphate-buffered saline (PBS) and stained with phalloidin to label F-actin, according to manufacturer’s instructions (Life Technologies). Briefly, samples were extracted with a permeabilization buffer of 0.1% Triton X-100 in PBS for 5 min, incubated with 1% bovine serum albumin in PBS for 30 min, and stained for 30 min with 6.6 *μ*M Alexa 488-phalloidin stain solution. Cell nuclei were stained with 300nM 4’,6-diamidino-2-phenylindole (DAPI) for 10 min. Epifluorescent images of random fields of view of single cells were acquired with a Nikon Eclipse TE2000-S microscope.

### Cell and nuclear morphology analysis

Fluorescent images were thresholded in NIH ImageJ to generate cell and nuclear outlines to analyze cell morphology parameters including spread area, Feret diameter, nuclear area, and nuclear aspect ratio.

### Analysis of cell-substrate migratory response

Cell trajectories were analyzed from timelapse micrographs with the Manual Tracking ImageJ plugin, using individual nuclei centroids for points of reference to measure displacement [16]. Directed migration was evaluated by calculating a durotactic index, the length a single cell migrates in the direction of increasing substrate stiffness divided by the total cell path [17, 18]. A larger index value indicates more biased motility in the direction of increased stiffness. For morphodynamics image analysis, cell outlines were manually traced from timelapse micrographs and analyzed for number of cellular processes projecting from the cell body, the length of the longest cell process, and the number of branch points per cell. Branching was determined by counting the number of points where a cellular process bifurcates into two discrete processes.

### Statistical analysis

All data are expressed as median ± median absolute deviation, unless otherwise noted. Quantitative data for cell morphology and behavior was first analyzed with Shapiro-Wilk normality tests to assess whether the data distribution was normal, p >*α* = 0.05. All reported data sets included at least one experimental group that was not normally distributed, therefore a non-parametric Kruskal-Wallis one-way ANOVA on ranks was used to statistically compare mean ranks and followed with Dunn’s multiple comparisons post-test. Significance was set at p <0.01. All results were collected from three independent experiments.

## Results

### PAA substrate characterization

**Fig. 1:**
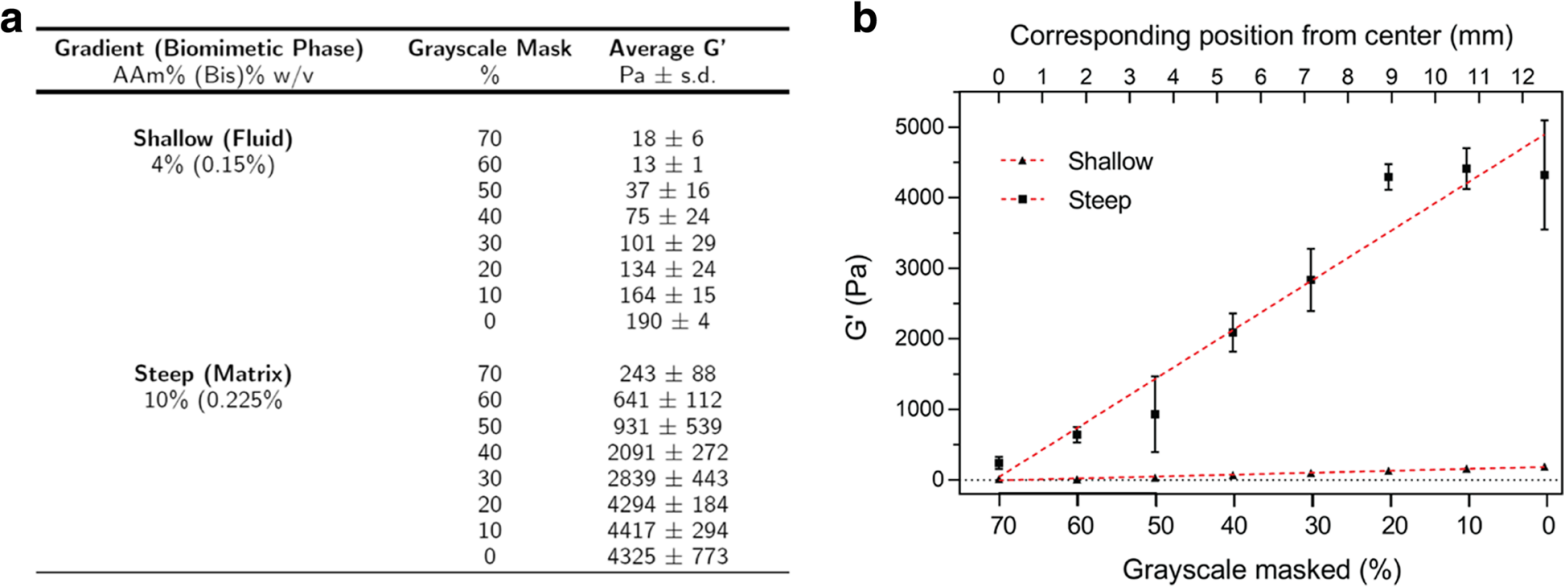
Mechanical characterization of PAA substrates. **a** Noted in the table are the percent concentrations of acrylamide (AAm) and bis-acrylamide (Bis) of the PAA substrates used in this study and the corresponding storage moduli G ^′^, measured by rheometry from the series of substrates UV polymerized with different percent grayscale masks. **b** The graph plots the same data, with percent grayscale masks, mapped to their corresponding sequential positions found on radial gradient substrates. Red dashed lines show the best fit linear regressions of data for the steep gradient (r^2^=0.940) and shallow gradient (r^2^=0.974). Black dotted line represents the equation y=0 for visual reference

For this study, we fabricated substrates tuned to recapitulate stiffnesses found within the mechanical niche of the peripheral nervous system (PNS) [3, 19, 20]. Shear storage moduli ranged from 18 ±6 Pa to 190 ±4 Pa for the shallow gradient and 243 ±88 to 4325 ±773 Pa for the steep gradient (Fig. 1). Nominal stiffness gradient slopes were approximated by performing linear regression on the data. For comparison with other studies that report gradient slope as a function of change in Young’s modulus over distance, the gradient slopes in this study correspond to 0.04 kPa/mm for the shallow gradient and 0.95 kPa/mm for the steep gradient. Rheology frequency plots are included in Additional file 1.

**Fig. 2:**
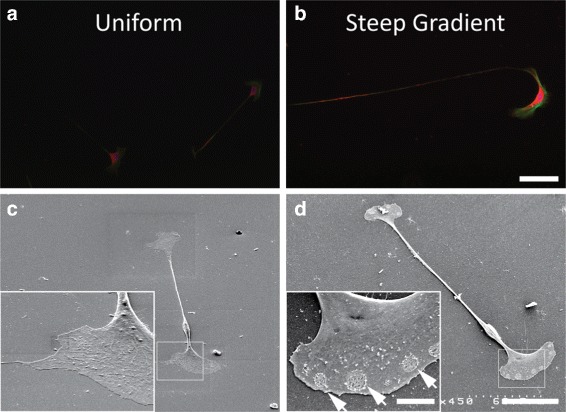
**a**, **b** Relative to Schwann cells cultured on **a** uniform substrates (4325 Pa), Schwann cells cultured on **b** steep gradient (243-4325 Pa) substrates had increased expression of paxillin (red), which co-localized to actin staining (green), indicating increased focal adhesion formation. Scale bar represents 100 *μ*m. **c**, **d** Scanning electron micrographs of Schwann cells adherent to substrates are shown at 450x magnification (scale bar is 50 *μ*m) and 20 degree tilt; inset 1500x magnification (scale bar is 10 *μ*m). Schwann cell on **c** uniform substrate exhibits intact plasma membrane surface. Schwann cell on **d** steep gradient exhibits plasma membrane morphology characterized by vesicles (arrows) fusing with the plasma membrane

Substrate surface characterization was performed to verify that mechanically uniform and gradient substrates were similar with respect to laminin ligand density and topography, two variables which can also influence Schwann cell phenotype [21, 22] and migration [23]. No difference in protein coating was observed either between substrates or across the length of gradient materials (Additional file 2). Similarly, SEM analysis of the cell-material interface between Schwann cells adherent to both uniform and gradient substrates revealed a homogeneous surface with no visible topographical differences between the substrate surfaces (Fig. 2). These observations indicated that Schwann cell behavior was not influenced by differences in either matrix ligand density or topography between the uniform and gradient substrates.

### Schwann cell morphology was insensitive to mechanically uniform substrates of different discrete stiffnesses

**Fig. 3:**
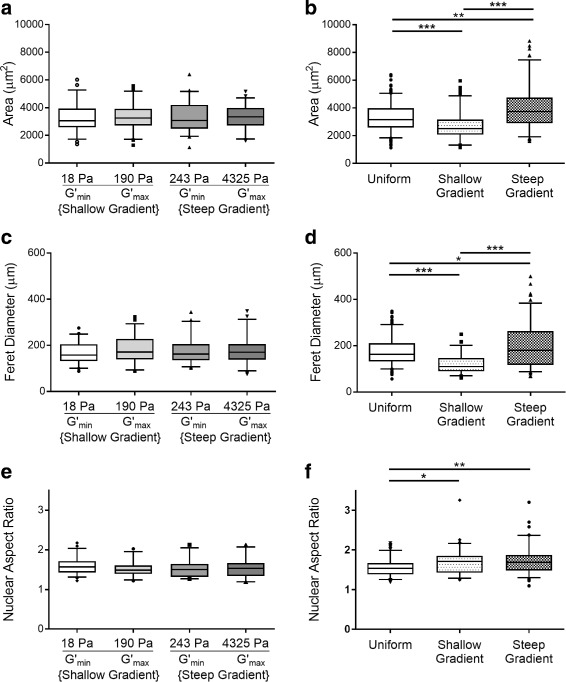
Schwann cell morphology was insensitive to increased stiffness across different mechanically uniform substrates. **a** Cellular spread area, **c** elongation (Feret diameter), and **e** nuclear aspect ratio were quantified for each substrate stiffness. Data is graphed with box (25^th^, median, and 75^th^ percentiles) and whisker (5^th^ and 95^th^ percentile) plots; dots are individual values beyond upper and lower 5th percentile. Relative to the morphologic cell response on uniform substrates, Schwann cells increased their **b** spread area and **d** elongation on the steep gradient substrates. In contrast, a decreased response was observed on the shallow gradient substrates. **f** Schwann cells elongated their nuclei on both gradient substrates, as indicated by increased nuclear aspect ratio. Statistical significance indicated by * for *p* <0.01, ** for *p* <0.001, and *** for *p* <0.0001, assessed by Kruskal-Wallis one-way ANOVA with Dunn’s post-test

We first analyzed morphologic response to substrate stiffness by culturing cells on four different mechanically uniform PAA substrates, with shear storage moduli corresponding to the minimum and maximum values of each of the two gradient substrates used later in this study (Fig. 1). When cultured on mechanically uniform substrates, Schwann cells had a similar morphologic phenotype across all stiffnesses tested. We observed no significant differences between Schwann cell spread area across uniform substrates of different stiffnesses. The median values for Schwann cell spread area, 3062 - 3333 *μ*m^2^, were comparable across all substrate groups tested (Fig. 3). Similarly, when we analyzed cell and nuclear elongation in Schwann cells on different uniform substrates, we observed no differences in Feret diameter, the greatest distance between any two points along a cell’s perimeter, nuclear aspect ratio, the ratio of the major to minor axes of a fitted ellipse around the nucleus, or nuclear area. Across substrates, median values ranged between 159.29 *μ*m and 174.99 *μ*m for Feret diameter and 1.33 and 1.43 for nuclear aspect ratio.

### Schwann cells altered their morphology in the presence of a steeper mechanical gradient

Qualitatively, Schwann cells cultured on the steep gradient substrate had a distinct morphologic phenotype compared to those cultured on the uniform substrates (Fig. 2). In Schwann cells adherent to the steep gradient substrate, we observed increased paxillin staining, which was co-localized to F-actin, indicating an increase in the formation of focal adhesions, which are necessary for migration. Upon ultrastructural analysis at high resolution using SEM, cell membranes were intact in cells on the uniform substrates. In contrast, in cells on the steep gradient, we observed plasma membrane perturbations, suggesting that lysosomal exocytic vesicles fused with the plasma membrane, a process previously correlated with lamellipodial extension [24] and post-injury remyelination [25]. On the steep gradient substrates, Schwann cells increased their spread area by 24% and elongation by 15% (Fig. 3). In contrast, Schwann cells cultured on shallow gradient substrates exhibited decreased spread area and cell elongation. The nuclear aspect ratio of cells was 1.711 ±0.199 and 1.687 ±0.185 in cells cultured on shallow and steep gradients respectively, both representing a modest increase in nuclear aspect ratio, compared to the median nuclear morphology in cells cultured on uniform substrates (1.541 ±0.129) (Fig. 3f).

### Schwann cell migration did not vary on mechanically uniform substrates of increasing stiffnesses

**Fig. 4:**
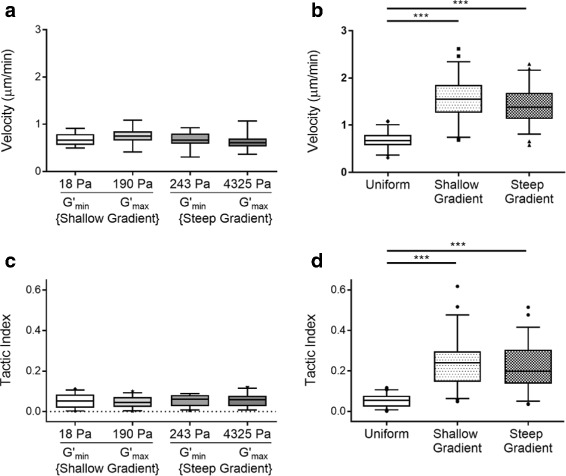
Schwann cell migration was similar across all mechanically uniform substrates evaluated. Data is graphed as box and whisker plots. No significant differences were found in Schwann cell **a** velocity or **c** tactic index, a metric of directedness calculated as the total cell path divided by the shortest distance between beginning and end position. Schwann cell migration on both gradient substrates was significantly **b** faster and **d** more directed than in cells adherent to uniform substrates. Statistical significance indicated by *** for p <0.0001, assessed by Kruskal-Wallis one-way ANOVA with Dunn’s post-test

Analysis of the averaged single cell velocities on the distinct mechanically uniform substrates evaluated in this study (18 Pa, 190 Pa, 243 Pa, 4325 Pa) indicated that cell speed did not vary as a function of substrate stiffness (Fig. 4). Similarly, the tactic index, a ratio of total cell trajectory divided by the length of the vector between a cell’s start and finish position, that serves as a metric of directed migration, was similar in cells adherent to the different uniform substrates evaluated.

### Schwann cell migration increased in speed and directedness on substrates with stiffness gradients

Median Schwann cell velocity on the uniform substrates was 0.67 *μ*m/min, whereas median velocities on the gradient materials were 1.55 *μ*m/min on the shallower gradient (231% increase), and 1.38 *μ*m/min on the steeper gradient (206% increase) (Fig. 4). Compared to the tactic index of cells cultured on the uniform substrates, Schwann cells on the shallow gradients displayed a 157% increase and those on the steep gradients similarly displayed a 151% increase.

### Schwann cells on gradient substrates extended and sustained more numerous, elongated, and branched cellular processes

**Fig. 5:**
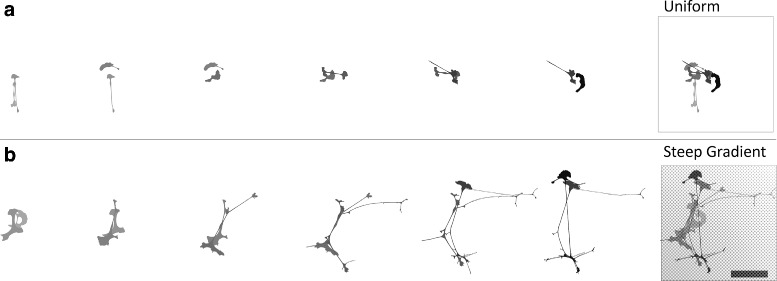
Overlaid cell boundaries from representative migrating Schwann cells on either **a** uniform or **b** steep gradient substrates. Left corresponds to earlier and right corresponds to later captured images and cell silhouettes are temporally color coded with cell shape demarcated in lighter grays for earlier time points and black for later time points. Frame interval is 2 hours. On the uniform substrate (4325 Pa), cell motion is random and morphology is dynamically sporadic, switching back and forth from a bipolar morphology to a more circular keratocyte-like shape. On the steep gradient substrate (190 - 4325Pa), note the increase in cell spread area and elongation, which is oriented along the direction of increasing stiffness on the rigidity gradient. Morphologic complexity is present throughout the duration of the timelapse. Boxed image: superimposed images from sequence at left. Scale bar represents 100 *μ*m

Qualitatively, Schwann cell morphology between cells on mechanically uniform and gradient substrates prominently differed throughout the entirety of the timelapse capture (Fig. 5). Initially on uniform substrates, most cells adopted a bipolar morphology, an observation supported by our prior work characterizing the biophysical response of Schwann cells to materials within a similar range of stiffness (∼0.24-4.8 kPa) [5]. Over time, as the cells displayed random walk motility on the uniform substrates, the dominant morphodynamic behavior observed was a fluctuation between extension into a bipolar morphology and retraction into either an asymmetric bipolar cell shape with a dominant leading edge or a curved keratocyte-like cell morphology. In contrast, most Schwann cells on the gradient substrates initially spread into a much more complex cell shape conformation, with multipolar morphology and strikingly extensive lamelipodia. Over time, cellular processes extended, oriented along the gradient toward a region of increased stiffness, followed by elongation and branching directed along the gradient. As the cell processes extended, the soma of the cell advanced directionally up the gradient, which was accompanied by a shift in localization of the leading edge of the cell, in front of the advancing nuclei.

**Fig. 6:**
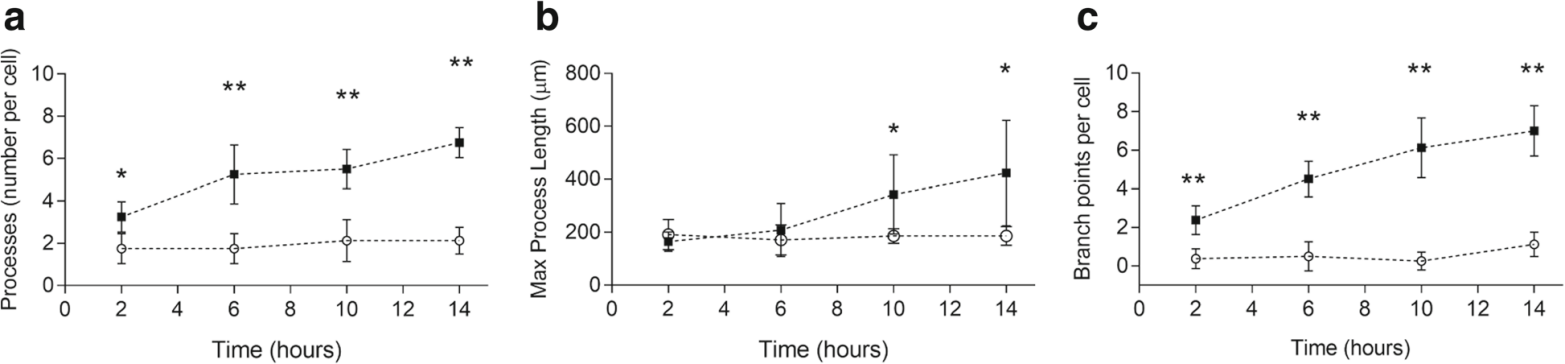
Morphodynamic behavior of Schwann cells cultured on uniform (4325 Pa) and steep gradient (190 - 4325 Pa) substrates. Schwann cells adherent to steep gradients (solid fill data points) extended and sustained more **a** numerous, **b** elongated, and **c** branched cellular processes than those cultured on mechanically uniform substrates (open fill data points). Significance noted with * for *p* <0.01 or ** for *p* <0.0001, determined by unpaired t-test. Values are means ± s.d

Although Schwann cells on the uniform materials displayed a static morphologic response over time, Schwann cells on gradient substrates exceeded those on uniform substrates in process number and branch points beginning at 2 hours, and in process length beginning at 10 hours in culture (Fig. 6). In contrast to cells on uniform materials, Schwann cells on the gradient materials increased their number, maximum process length, and branching over time.

## Discussion

### We evaluated Schwann cell behavior on substrates with stiffness gradients that recapitulated temporal mechanical niches of the PNS environment

The term physiologically relevant in referencing stiffness gradients has been previously defined as gradients that represent an innate range of stiffness expected in healthy tissue, approximately 1 kPa/mm or below [26]. However, physiologically relevant stiffness gradients will vary between specific cell types, given that each cell type exhibits a lineage-specific phenotype, influenced by its resident tissue niche and the temporal mechanical niche, which can vary dependent upon the relevant physiologic state. In this study, we evaluated Schwann cell morphology and migration in the presence of biomimetic stiffness gradients, designed to recapitulate the temporal mechanical niches present as repair state Schwann cells migrate across nerve defects. The two gradients correspond to two phases of the successful regeneration process that can occur in the PNS. Specifically, the shallow gradient corresponds to the initial fluid phase, in which plasma and extracellular matrix precursor molecules occupy the nerve defect, whereas the steep gradient recapitulates the subsequent matrix phase, in which a fibrin cable forms across the injury site to provide directional guidance for Schwann cells as they migrate into the nerve defect [27, 28]. Plasma and fibrin, the relevant biopolymers present during the fluid and matrix phases of PNS regeneration, have been previously characterized with rheometry, utilizing physiologically relevant multiaxial loading conditions that mimic in vivo loading conditions. The shear storage moduli of the substrates in our study (18-190 Pa for shallow gradient; 243-4325 Pa for steep gradient) align closely with previously reported values for plasma (∼15-160 Pa) and fibrin (∼400-5000 Pa), the primary constituent biopolymers within the respective fluid and matrix phase environments [29].

### Cells may be more sensitive stiffness gradient detectors than previously believed

**Fig. 7:**
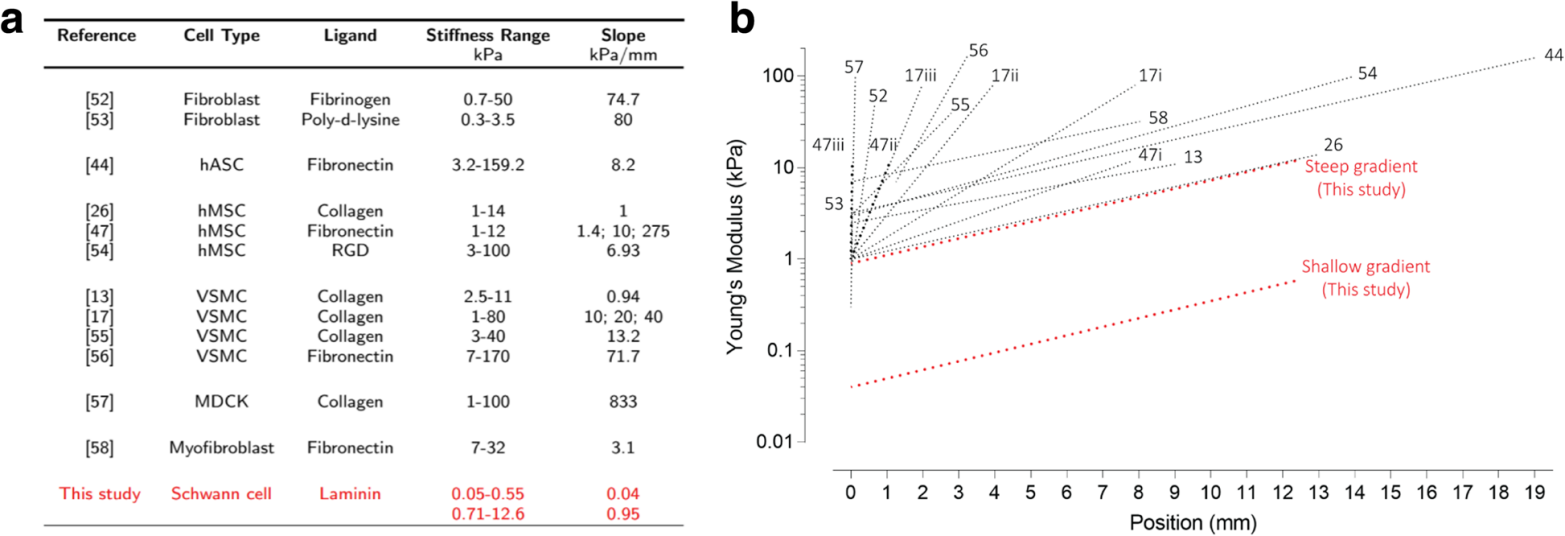
Summary of in vitro studies in which durotactic behavior was observed. **a** Table reports experimental parameters of the included studies, which used 2D substrates with continuous stiffness gradients as experimental platforms. **b** Graphical representation of studies listed in adjacent table. For comparison, each gradient is represented as an averaged linear slope across the entire length of the experimental substrate. Numbers on graph correspond to reference citations

In addition to providing the first evidence for Schwann cell durotaxis, our study is novel in demonstrating durotaxis in response to a stiffness gradient of such a shallow slope, specifically a change in stiffness per unit length an order of magnitude less than found in previous studies (Fig. 7). While the current understanding of durotaxis is far from complete, the results of this study suggest that Schwann cells are incredibly sensitive in detecting mechanical gradients. Our study raises the question of what the lower limit of stiffness gradient detection may be in other adherent cells. As other studies have suggested that cells are mechanically tuned to their native tissue environment, the specific magnitude of stiffness gradients at which other cells can propagate a durotactic response will require follow-up investigation [30].

### Morphologically, Schwann cells displayed a unique mechanophenotype

In several other cell types, including astrocytes and microglia, it has been found that substrate stiffness can regulate cell and nuclear morphology. As a function of increased substrate stiffness, most adherent cells increase their spread area [30–32], length [33, 34], and nuclear aspect ratio [35, 36]. In contrast to the cell-material response typically observed in other mesenchymal cells, our results suggest that Schwann cell morphology is not variable as a function of increased absolute substrate stiffness, within our physiologically relevant stiffness range tested (G ^′^= 18 - 4325 Pa) (Fig. 3).

Following the observation that Schwann cell morphology was similar across substrates tuned to different discrete moduli, we next investigated whether Schwann cells would alter cellular and nuclear morphology in response to a stiffness gradient. It is known that following injury Schwann cells navigate across the mechanically heterogeneous nerve defect and transdifferentiate into a pro-regenerative phenotype. These specialized repair Schwann cells are characterized by increased cell area and elongation. Informed by the in vivo scenario, we hypothesized that substrates with embedded stiffness gradients could elicit the pro-regenerative morphology in Schwann cells. To test this hypothesis, we evaluated Schwann cell morphology on substrates embedded with the aforementioned shallow and steep gradients, respectively corresponding to the fluid and matrix PNS regeneration phases.

Quantitative assessments of Schwann cell morphology on both gradient substrate conditions, compared to the combined data of cell response on uniform substrates revealed that Schwann cells displayed a pro-regenerative morphologic phenotype in response to the steep gradient only. Our results suggest that there may exist a threshold mechanical gradient steepness capable of eliciting the pro-regenerative morphological response in Schwann cells. The morphologic response that we observed in Schwann cells on the steep gradient substrates resembles the dynamic response of repair state Schwann cells in vivo, which transiently are altered transcriptionally to transform into a pro-regenerative repair state, characterized by a morphologically distinct phenotype [11, 37]. This morphologic specialization is critical as these cells collectively assemble into bands of Büngner, structures composed of aligned and elongated Schwann cells, functionally serving as tracks which guide axonal regrowth back towards their innervation target [38].

In contrast to the differential cellular morphologic response to the shallow and steep gradients, Schwann cell nuclear morphology was comparable when Schwann cells were cultured on either gradient substrate. The increased nuclear aspect ratio indicated that Schwann cells adopted a more elongated nuclear morphology in the presence of a mechanical gradient. The fact that the cellular and nuclear morphologic responses to mechanical gradients of varying slopes were not parallel suggests that Schwann cells may engage in multiple mechanisms of mechanotransduction in the presence of mechanical gradients, as has been observed previously in other instances of mechanotransduction [39].

### Schwann cells can migrate via durotaxis

Before evaluating cell response to substrates embedded with stiffness gradients, we first investigated whether Schwann cell migration varied on mechanically uniform substrates of different stiffnesses. Experiments were carried out at low seeding density to minimize cell-cell interactions, to better isolate the cell-material response. Our results suggested that, within the physiologically relevant stiffness range tested, Schwann cells do not alter their migratory phenotype as a function of substrate stiffness. In contrast to the results observed here, a previous study reported higher Schwann cell velocities in response to more compliant substrate stiffnesses [4]. Both studies employed laminin-coupled PAA platforms within similar mechanical ranges (0.04-12.6 kPa in our study compared to 1-20 kPa). However, several experimental differences could account for the incongruent results, including extracellular matrix ligand density (200 *μ*g/mL laminin in our study compared to 10 *μ*g/mL), substrate surface chemistry (covalent crosslinking in our study compared to physical adsorption), and cell seeding density (sparse in our study compared to relatively dense). Each of these variables has been previously demonstrated to influence cell migration [40–44]. Given that cell motility observed in several other cells including fibroblasts [45], vascular smooth muscle cells [23], and neutrophils [46], is dependent upon substrate stiffness, our observation that Schwann cell migration was not variable on substrates of different uniform stiffnesses was unexpected. However, this finding was consistent with our observation that Schwann cell morphology was insensitive to substrate stiffness within the mechanical range tested (Fig. 3). Future studies will be necessary to elucidate the covariable effects of matrix stiffness, ligand density, and cell density on Schwann cell migratory phenotype.

When we next posed the question whether cell migration would change in response to a mechanical gradient, we observed that Schwann cells showed increased speed on both gradients, in comparison to cells cultured on uniform substrates. Further, Schwann cells on both gradients had an increased tactic index, indicating a more directed migratory path, relative to cells on the uniform substrates. The data was notable in showing that Schwann cell migration was similar on the different gradient substrates evaluated. This observation suggests that the presence of the gradient, independent of its slope, can generate a durotactic response in Schwann cells. This finding adds to the growing body of literature suggesting that durotactic behavior is cell-type specific, in providing a counterpoint example to other adherent cells in which durotaxis was dependent upon gradient strength [17, 47]. Considering the unique migratory behavior displayed by Schwann cells in our study, our results suggest that the role of mechanical gradient steepness on durotaxis may be more complex than previously thought and specific to the native tissue niche where specific cells reside.

### Stiffness gradients may expedite morphologic specialization in Schwann cells

The effect of stiffness gradients on cell morphology over time remains an open question. Most durotaxis studies analyze directional cell migration in response to a stiffness gradient through tracking the translocation of a cell’s nuclear centroid, irrespective of morphologic changes in cellular processes extending from the soma. However, given the critical relationship between cell morphology and function in cells of the nervous system, there is a need to better understand how the cellular mechanical microenvironment contributes to temporal changes in cell morphology during both developmental and regenerative processes. Along with a growing body of evidence that mechanical stimuli can increase the rate of lamelliodial extension [24], multiple studies report a variety of effects of increased stiffness on cellular process dynamics in different resident cells of the nervous system [32, 48–51].

Following up on our observation that Schwann cells were more elongated on the steep gradient substrates, we analyzed Schwann cell morphodynamics on the steep gradient relative to that observed on uniform substrates to better understand this temporal process. Specifically, we examined the number, maximum process length, and branching of cellular processes, hypothesizing that these morphologic metrics would be greater on the gradient substrates. Our results indicate that Schwann cells, within 2 hours of contact with a stiffness gradient, can display a morphology similar to the highly elongated and branched morphology of repair state Schwann cells, a morphology which does not appear until several weeks following injury in vivo. This observation suggests that durotactic material grafts may expedite morphologic specialization. This finding is important considering that Schwann cells function in multiple ways during the regenerative process to collectively contribute to proper axonal regrowth. Initially, directed migration of Schwann cells into the nerve defect is critical. Subsequently, it is imperative that these cells also elongate to provide a guiding scaffold for regenerating axons to traverse. A biophysical stimulus, such as a stiffness gradient, may offer a novel effective biomaterial cue, providing sustained directional guidance in the post-nerve injury environment, through influencing both the position and extended morphology of Schwann cells across a nerve defect.

## Conclusion

Overall, our study reveals that Schwann cells can migrate via durotaxis and provides insight into the inherent and unique mechanosensitivity of these cells, which is divergent from previous reports of durotaxis in other adherent cell types. Specifically, whereas in other cell types, durotaxis has been dependent upon the gradient slope, Schwann cells in our study demonstrated directed migration in response to the presence of a stiffness gradient, independent of its magnitude. Although a pro-migratory response was seen in cells on both gradient substrates, we only observed a paired pro-regenerative phenotype in cells cultured on the steep gradient substrates, an observation that parallels the in vivo corollary regenerative phases. During the fluid phase, modeled by the shallow gradient, Schwann cells are migrating towards the nerve injury site, whereas during the matrix phase, modeled by the steep gradient, the cells continue to migrate across the injury site and simultaneously elongate, serving as guiding scaffolding for regenerating axons.

Informing neural tissue engineering, our results suggest that embedding stiffness cues into a bioengineered nerve graft presents another promising strategy to control Schwann cell migration following injury to ultimately promote successful nerve regeneration. Future studies will be required to evaluate whether this migratory phenomena can be induced in repair Schwann cells in vivo through implanting durotactically engineered nerve guidance channels. Further, the potentially expedited process of directed migration of Schwann cells towards forming bands of Büngner much earlier in the wound healing process is a promising technique for promoting nerve regeneration, potentially capable of bridging larger nerve defects. More broadly, there exist several tissue engineering applications for durotactic biomaterials to treat various pathologies of the central nervous system, through directed cell migration.

## Additional files

Additional file 1 Rheological characterization of PAA gels that approximate sequential regions on the experimental gradients. Graphs of storage moduli versus angular frequency, for substrates which correspond to eight distinct positions on each of the **a.** shallow and **b.** steep gradients. Substrates vary in stiffness as a function of the amount of UV light transmitted through different grayscale masks to polymerize the pre-polymer PAA solutions. Individual plots correspond to grayscale masks (0-70%, steps of 10%). n=3-6 for each substrate condition. For numeric average values ± s.d., refer to Fig. 1 in text. (PDF 129 kb)

Additional file 2 Similar amounts of laminin were present on uniform and gradient substrates. **a.** Fluorescent micrographs of PAA substrates with covalently bound laminin, immunostained with anti-laminin primary antibody and Cy3-conjugated secondary antibody. **b.** Relative fluorescence is reported in arbitrary units and graphed as mean ± s.d. Data shown for uniform (4325 Pa) and steep gradient substrates (243- 4325 Pa). Scale bar represents 200 *μ*m. (PDF 84.7 kb)

## Abbreviations

AAm: Acrylamide
Bis: Bis-acrylamide
DAPI: 4’,6-diamidino-2-phenylindole
PAA: Polyacrylamide
PBS: Phosphate-buffered saline
PNS: Peripheral nervous system
SEM: Scanning electron microscope
