# Supplementary figures and images for: Schwann cell durotaxis can be guided by physiologically relevant stiffness gradients
Source: Biomater Res. 2018 May 9;22:14. doi: 10.1186/s40824-018-0124-z (PMC5948700; doi:10.1186/s40824-018-0124-z)

## Additional File 1

File name: Additional\_File\_1.png

**a.**

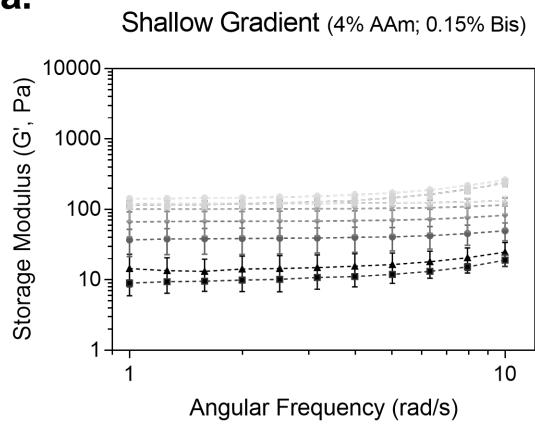

**b.**

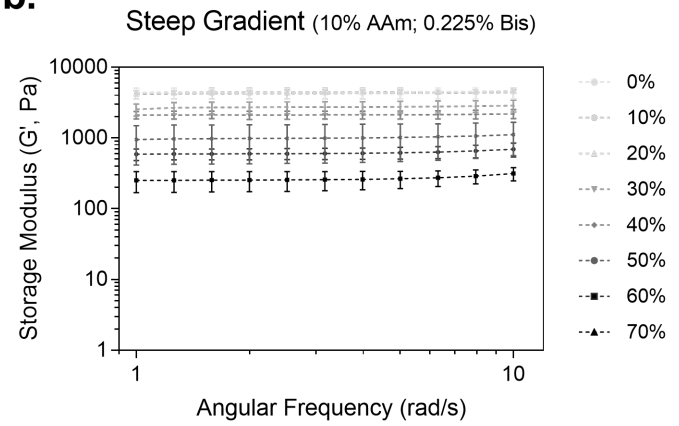

Supplement: Supplementary file 1 — Rheological characterization of PAA gels that approximate sequential regions on the experimental gradients. Graphs of storage moduli versus angular frequency, for substrates which correspond to eight distinct positions on each of the a. shallow and b. steep gradients. Substrates vary in stiffness as a function of the amount of UV light transmitted through different grayscale masks to polymerize the pre-polymer PAA solutions. Individual plots correspond to grayscale masks (0-70%, steps of 10%). n=3-6 for each substrate condition. For numeric average values ± s.d., refer to Fig. 1 in text. (PDF 129 kb) [file 40824_2018_124_MOESM1_ESM.pdf]

## Additional File 2

File name: Additional\_File\_2.png

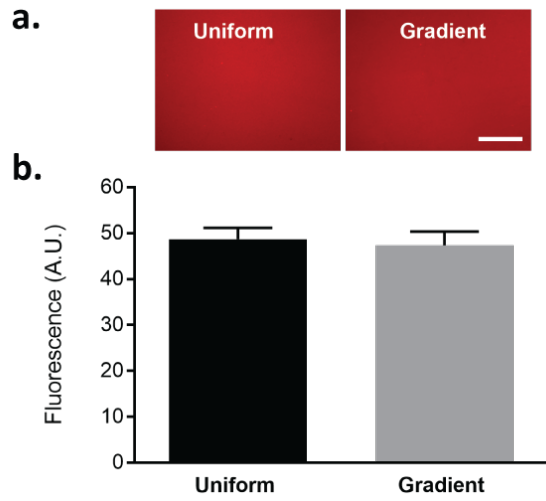

Supplement: Supplementary file 2 — Similar amounts of laminin were present on uniform and gradient substrates. a. Fluorescent micrographs of PAA substrates with covalently bound laminin, immunostained with anti-laminin primary antibody and Cy3-conjugated secondary antibody. b. Relative fluorescence is reported in arbitrary units and graphed as mean ± s.d. Data shown for uniform (4325 Pa) and steep gradient substrates (243- 4325 Pa). Scale bar represents 200 μm. (PDF 84.7 kb) [file 40824_2018_124_MOESM2_ESM.pdf]
